# Team Objective Structured Bedside Assessment (TOSBA) as formative assessment in undergraduate Obstetrics and Gynaecology: a cohort study

**DOI:** 10.1186/s12909-015-0456-5

**Published:** 2015-10-09

**Authors:** Richard P. Deane, Pauline Joyce, Deirdre J. Murphy

**Affiliations:** 1 Department of Obstetrics & Gynaecology, Trinity College, University of Dublin, Coombe Women & Infants University Hospital, Dublin 8, Dublin, Republic of Ireland; 2 School of Medicine, Royal College of Surgeons in Ireland, 123 St Stephen’s Green, Dublin 2, Dublin, Republic of Ireland

**Keywords:** Team objective structured bedside assessment, Formative assessment, Clinical skills, Reasoning, Obstetrics & gynaecology, Academic performance

## Abstract

**Background:**

Team Objective Structured Bedside Assessment (TOSBA) is a learning approach in which a team of medical students undertake a set of structured clinical tasks with real patients in order to reach a diagnosis and formulate a management plan and receive immediate feedback on their performance from a facilitator. TOSBA was introduced as formative assessment to an 8-week undergraduate teaching programme in Obstetrics and Gynaecology (O&G) in 2013/14. Each student completed 5 TOSBA sessions during the rotation. The aim of the study was to evaluate TOSBA as a teaching method to provide formative assessment for medical students during their clinical rotation. The research questions were: Does TOSBA improve clinical, communication and/or reasoning skills? Does TOSBA provide quality feedback?

**Methods:**

A prospective cohort study was conducted over a full academic year (2013/14). The study used 2 methods to evaluate TOSBA as a teaching method to provide formative assessment: (1) an online survey of TOSBA at the end of the rotation and (2) a comparison of the student performance in TOSBA with their performance in the final summative examination.

**Results:**

During the 2013/14 academic year, 157 students completed the O&G programme and the final summative examination . Each student completed the required 5 TOSBA tasks. The response rate to the student survey was 68 % (*n* = 107/157). Students reported that TOSBA was a beneficial learning experience with a positive impact on clinical, communication and reasoning skills. Students rated the quality of feedback provided by TOSBA as high. Students identified the observation of the performance and feedback of other students within their TOSBA team as key features. High achieving students performed well in both TOSBA and summative assessments. The majority of students who performed poorly in TOSBA subsequently passed the summative assessments (*n* = 20/21, 95 %). Conversely, the majority of students who failed the summative assessments had satisfactory scores in TOSBA (*n* = 6/7, 86 %).

**Conclusions:**

TOSBA has a positive impact on the clinical, communication and reasoning skills of medical students through the provision of high-quality feedback. The use of structured pre-defined tasks, the observation of the performance and feedback of other students and the use of real patients are key elements of TOSBA. Avoiding student complacency and providing accurate feedback from TOSBA are on-going challenges.

## Background

Assessment can be formative (guiding future learning, providing reassurance, promoting reflection shaping values) or summative (making an overall judgment about competence, fitness to practice, or qualification for advancement to higher levels of responsibility) [1]. The role of summative assessment is well established in medical schools. However, the role of formative assessment is less defined but its benefit is increasingly recognised [2–4]. A wide range of tools to assess clinical skills of medical students have been described in the literature [5]. However, there has been little evaluation of their use as formative assessment. The use of specific teaching methods to provide formative assessment in the clinical environment may address some of the drawbacks traditionally associated with clinical teaching including unclear objectives, unstructured approaches and poor learner feedback [6]. Team Objective Structured Bedside Assessment (TOSBA) is a method of formative assessment involving a series of bedside encounters with real hospital patients [7, 8]. Each encounter requires a ‘team’ of students to undertake a set of key clinical tasks with facilitator feedback. TOSBA aims to facilitate learning of key clinical, communication and reasoning skills. TOSBA may address some of the drawbacks associated with clinical teaching highlighted previously [6]. However, despite its intuitive potential to provide beneficial formative assessment, TOSBA has only been evaluated to date in a single institution in the disciplines of medicine and surgery and requires further evaluation.

The aim of the study was to evaluate TOSBA as a teaching method to provide formative assessment for medical students during their Obstetrics & Gynaecology (O&G) clinical rotation. The key elements of formative assessment are *the provision of quality feedback* in order *to enable learning* [9]. In order to achieve the aim of this study, the following research questions considered: Does TOSBA improve clinical, communication and/or reasoning skills? Does TOSBA provide quality feedback?

## Methods

### Study setting

The study was undertaken within the Department of Obstetrics & Gynaecology at Trinity College, University of Dublin.

#### Teaching

The undergraduate programme in Obstetrics & Gynaecology (O&G) is an 8-week rotation during the penultimate year of the 5-year course in medicine. There are 4 rotations during the academic year with approximately 40 students in each rotation. The class is divided evenly into 4 groups in advance by the medical school and each group completes 1 of 4 separate rotations during the year (Sep/Oct, Nov/Jan, Feb/Mar and Apr/May). Students complete an intensive programme of classroom-based and clinical activities during their rotation. TOSBA was not a component of the teaching programme in O&G or any other discipline in the medical school prior to the study. Each student is required to complete a logbook during the rotation and submit it to the Department at the end of the rotation. The importance of student attendance is emphasised throughout the rotation.

#### Assessment

The overall examination score for the undergraduate programme in O&G is determined by an end-of-rotation Objective Structured Clinical Examination consisting of 3 stations (OSCE, 25 %), an end-of-year written examination consisting of 50 single best answer questions (SBA, 10 %), short answer questions consisting of 6 clinical scenarios (SAQ, 30 %) and an end-of-year long case clinical examination (LCCE, 35 %). The University requires the use of a fixed standardised marking scheme across all faculties including medicine. Students require an overall examination score of 50 % or more to pass the examination. In addition, students must pass the LCCE to pass the examination regardless of their performance in any other component. Students achieving an overall examination score of 60 % or more are awarded a distinction (‘Honours’). Criterion referenced standard setting is applied to each component of the examination. This results in variable pass marks across the different examination components. The raw scores obtained by students in each examination component are then converted to the fixed standardised marking scheme.

### TOSBA

**Fig. 1:**
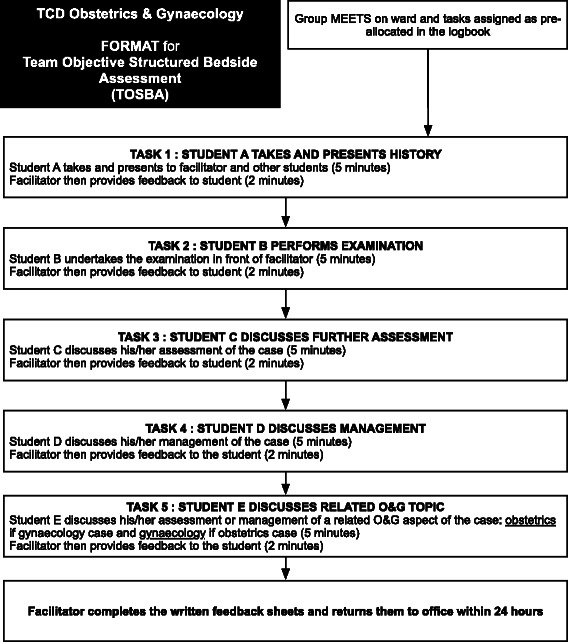
TOSBA format

Each TOSBA session required a ‘team’ of 5 students to undertake a set of pre-defined tasks on each patient: take a history, perform a physical examination, formulate a differential diagnosis, formulate a management plan and discuss a related O&G aspect of the case (obstetrics if a gynaecology case and gynaecology if an obstetrics case) (Fig. 1). Each task was undertaken by an individual student under the observation of a facilitator who provided immediate verbal feedback at the end of that task. Each task was allocated 7 min: 5 min for the student to complete the task and 2 min for the facilitator to provide verbal feedback. Therefore, each patient encounter lasted approximately 30 min. Each task built on previous tasks i.e. to formulate a differential diagnosis required the successful taking of the patient’s history and performance of a physical examination. Therefore, the ‘team’ element of TOSBA refers to the fact that in order for a student to complete a particular task, the successful completion of previous tasks by different students was required.

**Fig. 2:**
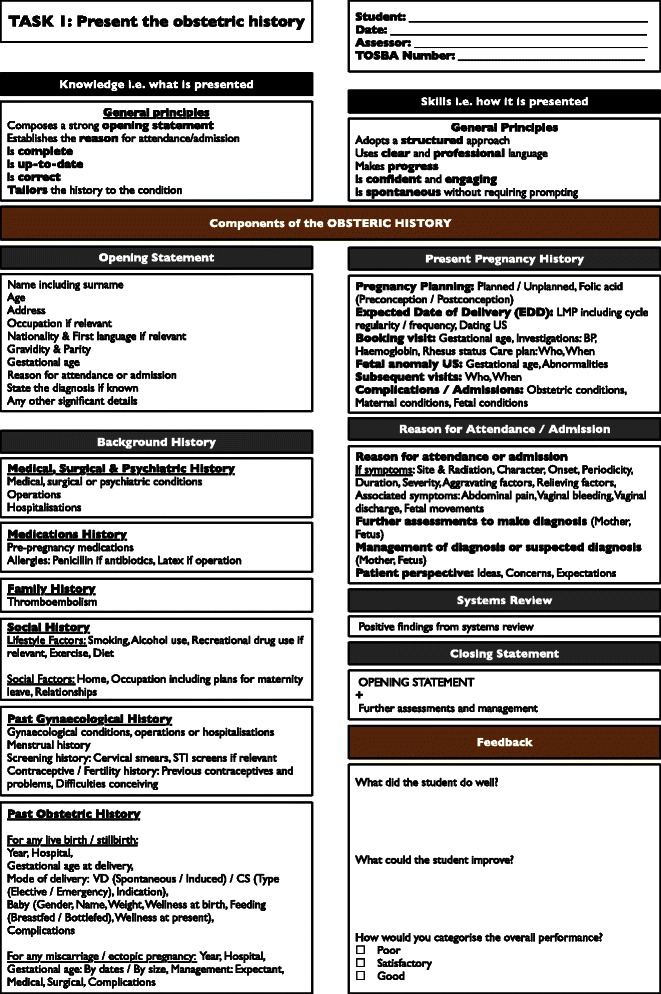
Example TOSBA feedback sheet

After the TOSBA session the facilitator completed a written feedback sheet for each student. A specific written feedback sheet was created for each task in order to provide consistency (Fig. 2). Each written feedback sheet required the facilitator to provide both qualitative and quantitative feedback. In terms of qualitative feedback, the facilitator provided free text comments on what the student did well and what the student could improve. In terms of quantitative feedback, the facilitator provided a rating of the student’s performance into 1 of 3 grades: poor demonstration of the task, satisfactory demonstration of the task or good demonstration of the task. The term ‘fail’ was not used in order to avoid demotivation. These grades did not contribute towards their end-of-rotation score and were used as feedback only i.e. formative and not summative. The facilitator then returned the completed feedback sheets to the administrative officer and students collected their feedback sheets directly from the administrative officer.

TOSBA was introduced as formative assessment to the undergraduate teaching programme in O&G during the 2013/14 academic year. A specific schedule of times, locations and facilitators for each TOSBA session was created in advance of each rotation. The tasks students were required to complete were also specified in advance. The sessions were incorporated into the student logbook and distributed on the first day of the rotation. Each team met with 5 patients in total (and therefore had 5 TOSBA sessions in total) during the rotation so that each student performed each task. Each team remained the same throughout the rotation although the individual tasks varied with each session. All students were required to participate in TOSBA as it was introduced as a compulsory component of the programme. An explanatory flowchart was prepared and distributed among students and facilitators at the start of the rotation to explain the format (Fig. 1). A core team of 5 staff members facilitated the TOSBA sessions. Each facilitator was a senior clinician in O&G (at senior registrar or consultant level) and a full-time academic member of the Department of O&G who had regular teaching sessions. Therefore, each facilitator was experienced at providing feedback to medical students. In addition, all facilitators met in advance of the academic year to agree the approach and format to providing feedback.

### Study design

**Fig. 3:**
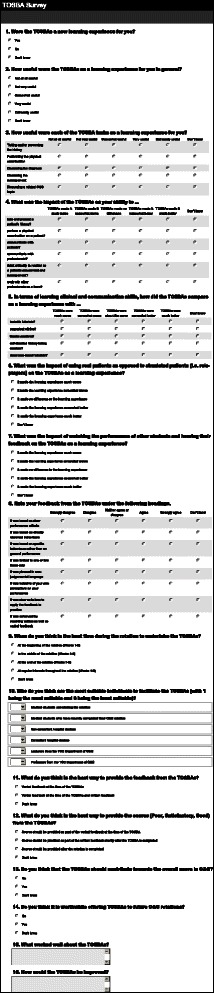
Student survey

The study was designed as a prospective cohort study over a full academic year (2013/14). The study used 2 approaches to evaluate TOSBA as a teaching method to provide formative assessment and address the study research questions. Firstly, students were asked to complete an online survey on TOSBA at the end of their rotation. A combination of quantitative and qualitative questions were used: 14 quantitative and 2 qualitative questions (Fig. 3). For quantitative questions 5-point Likert scales were used with an additional ‘don’t know’ option. For qualitative questions free text boxes were used. In relation to the quality of feedback, students were asked to rate their feedback based on a number of criteria that had been identified in the literature as important in order to provide effective feedback [10]. Secondly, student performance in TOSBA was compared with their performance in the summative examination. The study received Institutional Research Ethics Board approval (Faculty of Medicine Research Ethics Committee, Trinity College Dublin, July 2013).

### Participants

All 158 students completing their O&G rotations in the September 2013 to June 2014 academic year were invited to complete the online survey. Students were given the opportunity to opt out of the survey and indicated consent by participating in the survey.

Students who did not complete the summative examination were excluded.

### Data collection

The survey was conducted online using a survey tool (Survey Monkey®). The responses to the survey were anonymous. The survey was sent by e-mail during the week following the end of each of the 4 rotations. The survey was sent to non-responders on 2 further occasions and any non-responders at that stage were not contacted further. Student demographic information (gender, age, nationality), TOSBA grades and summative examination scores were obtained from departmental records.

### Data analysis

#### Analysis of student survey

Descriptive statistics were used to describe student responses to the quantitative questions on the survey. Not all respondents answered every question and therefore the number of responses to each question is stated in the results section. Student responses to qualitative questions were analysed using content analysis for themes. ATLAS.ti version 7 was used to assist content analysis. The profile of responders to the students survey (gender, age, nationality) was compared with non-responders using the Chi-squared and Fisher’s exact tests.

#### Analysis of TOSBA grades and summative examination scores

Descriptive statistics were used to describe TOSBA grades and summative examination scores. Students were classified into 1 of 4 TOSBA categories based on their 5 TOSBA grades as follows:

- ‘Overall Excellent’ if they achieved ≥3 ‘Good’ grades
- ‘Overall Good’ if they achieved ≥3 ‘Satisfactory’ grades and ≥1 ‘Good’ grade
- ‘Overall Satisfactory’ if they achieved ≥3 ‘Satisfactory’ grades but no ‘Good’ grades or if they did not achieve ≥3 of the same grade
- ‘Overall Poor’ if they achieved ≥3 ‘Poor’ grades

It should be emphasised that students received their TOSBA grade (i.e. Good, Satisfactory, Poor) for each task as it was completed but did not receive their overall TOSBA category as this was calculated after the rotation as part of the data analysis for this study. TOSBA performance categories were compared with summative examination performance categories using the Chi-squared and Fisher’s exact tests. SPSS version 22 was used for statistical analysis. A *p*-value of 0.05 was used for statistical significance.

## Results

### Participants

During the 2013/14 academic year, 158 students completed the O&G programme: 38 students in rotation 1 (24 %), 38 students in rotation 2 (24 %), 41 students in rotation 3 (26 %) and 41 students in rotation 4 (26 %). Each student completed the required 5 TOSBA tasks i.e. 158 students completed 790 TOSBA tasks. A student from rotation 3 was absent from the summative examination and was excluded from the analysis. The response rate to the student survey was 68 % (*n* = 107/157). There was no difference between responders and non-responders in terms of age (*p* = 0.573) or nationality (*p* = 0.550). However, female students were more likely to respond than male students (*p* = 0.004). A demographic profile is provided in Table 1.

**Table 1:** Student demographic profile

	All Students (*n =* 157)	Students who responded to the survey (*n* = 107)	Students who did not respond to the survey (*n* = 50)	*P* value
Gender				
Male	77 (49 %)	44 (41 %)	33 (66 %)	0.004
Female	80 (51 %)	63 (59 %)	17 (34 %)	
Age				
20–24 years	121 (77 %)	85 (79 %)	36 (72 %)	0.573
25–29 years	25 (16 %)	15 (14 %)	10 (20 %)	
≥30 years	11 (7 %)	7 (7 %)	4 (8 %)	
Nationality				
Irish	103 (66 %)	74 (69 %)	29 (58 %)	0.550
North American	22 (14 %)	15 (14 %)	7 (14 %)	
Asian	16 (10 %)	9 (8 %)	7 (14 %)	
British	7 (4 %)	4 (4 %)	3 (6 %)	
Other EU	5 (3 %)	2 (2 %)	3 (6 %)	
African	4 (3 %)	3 (3 %)	1 (2 %)	

### Student survey – quantitative questions

TOSBA was a new experience for nearly all students (*n* = 106/107, 99 %). The quantitative questions addressed 3 aspects of TOSBA: the learning provided by TOSBA, the feedback provided by TOSBA and the format of TOSBA.

#### Learning provided by TOSBA

Table 2 summarises the responses to the questions addressing the learning provided by TOSBA. The majority responded that it would be worthwhile offering TOSBA to future O&G rotations (*n* = 104/107, 97 %) with only a small number responding ‘don’t know’ (*n* = 3/107, 3 %) or ‘no’ (*n* = 0/107, 0 %).

**Table 2:** Learning provided by TOSBA – student survey responses

How useful were the TOSBAs as a learning experience for you in general?						
	Don’t know	Not at all useful	Not very useful	Somewhat useful	Very useful	Extremely useful
	2 (2 %)	0 (0 %)	0 (0 %)	10 (10 %)	42 (40 %)	51 (48 %)
How useful were each of the TOSBA tasks as a learning experience for you?						
	Don’t know	Not at all useful	Not very useful	Somewhat useful	Very useful	Extremely useful
Taking and/or presenting history	0 (0 %)	0 (0 %)	2 (2 %)	12 (11 %)	34 (34 %)	59 (50 %)
Performing the examination	0 (0 %)	0 (0 %)	4 (4 %)	13 (12 %)	37 (35 %)	53 (49 %)
Discussing the diagnosis	0 (0 %)	0 (0 %)	1 (1 %)	13 (12 %)	35 (33 %)	57 (54 %)
Discussing the management	0 (0 %)	0 (0 %)	2 (2 %)	8 (7 %)	37 (35 %)	60 (56 %)
Discussing a related O&G topic	0 (0 %)	2 (2 %)	12 (11 %)	33 (31 %)	38 (35 %)	22 (21 %)
What was the impact of the TOSBAs on your ability to?						
	Don’t know	Much worse	Somewhat worse	About the same	Somewhat better	Much better
Take and/or present a history	0 (0 %)	0 (0 %)	1 (1 %)	8 (7 %)	56 (52 %)	42 (40 %)
Perform an examination	0 (0 %)	1 (1 %)	0 (0 %)	13 (12 %)	49 (46 %)	44 (41 %)
Communicate with patients	0 (0 %)	0 (0 %)	1 (1 %)	57 (53 %)	32 (30 %)	17 (16 %)
Communicate with professionals	0 (0 %)	0 (0 %)	1 (1 %)	22 (21 %)	43 (40 %)	41 (38 %)
Think critically about patients	1 (1 %)	0 (0 %)	0 (0 %)	4 (4 %)	40 (38 %)	61 (57 %)
Work with other professionals	1 (1 %)	0 (0 %)	2 (2 %)	42 (40 %)	39 (36 %)	23 (21 %)
In terms of learning clinical and communication skills, how did the TOSBAs compare as a learning experience with?						
	Don’t know	Much worse	Somewhat worse	About the same	Somewhat better	Much better
Bedside Tutorials	1 (1 %)	1 (1 %)	8 (7 %)	35 (33 %)	53 (50 %)	9 (8 %)
Outpatient Clinics	0 (0 %)	1 (1 %)	3 (3 %)	7 (6 %)	29 (27 %)	67 (63 %)
Theatre Sessions	0 (0 %)	1 (1 %)	7 (6 %)	12 (11 %)	35 (33 %)	52 (49 %)
Self-Directed Work	0 (0 %)	0 (0 %)	3 (3 %)	6 (6 %)	30 (28 %)	68 (63 %)
Classroom Tutorials	0 (0 %)	4 (4 %)	3 (3 %)	16 (15 %)	51 (47 %)	33 (31 %)

#### Feedback provided by TOSBA

Table 3 summarises the responses to the questions addressing the feedback provided by TOSBA.

**Table 3:** Feedback provided by TOSBA in rank order – student survey responses

Rate your feedback from the TOSBAs under the following headings.								
*It was …*	Don’t know	Strongly disagree	Disagree	Neither agree nor disagree	Agree	Strongly agree	Score *M SD R*	
Phrased in non-judgemental language	8 (8 %)	1 (1 %)	3 (3 %)	18 (17 %)	44 (42 %)	31 (29 %)	4.04 0.85 0–5	1
Based on directly observed behaviours	11 (11 %)	0 (0 %)	5 (5 %)	10 (10 %)	62 (59 %)	16 (15 %)	3.96 0.71 0–5	2
Based on clear performance criteria	14 (13 %)	1 (1 %)	6 (6 %)	17 (16 %)	52 (50 %)	15 (14 %)	3.81 0.83 0–5	3
Clear as to how to apply feedback in practice	7 (7 %)	0 (0 %)	11 (11 %)	16 (15 %)	53 (50 %)	18 (17 %)	3.80 0.87 0–5	4
Based on specific behaviours	16 (16 %)	0 (0 %)	14 (14 %)	30 (29 %)	34 (33 %)	8 (8 %)	3.42 0.87 0–5	5
Enhanced by receiving written feedback	16 (16 %)	7 (7 %)	19 (18 %)	18 (17 %)	26 (25 %)	17 (17 %)	3.31 1.24 0–5	6
Inclusive of students’ own perceptions	13 (13 %)	3 (3 %)	22 (21 %)	20 (19 %)	40 (38 %)	6 (6 %)	3.26 1.01 0–5	7
Limited to 1–2 items only	14 (14 %)	0 (0 %)	28 (27 %)	25 (24 %)	26 (25 %)	10 (10 %)	3.20 1.01 0–5	8

The score mean (*M*), standard deviation (*SD*) and range (*R*) were calculated from ‘strongly disagree’ (1) to ‘strongly agree’ (5), excluding ‘don’t knows’

### Format of TOSBA

- Students ranked the lecturers and professors from the Department as *the most suitable individuals to facilitate TOSBA and provide feedback* with the following mean rankings (standard deviation, range) from 1 (being the most suitable) to 6 (being the least suitable): 2.6 (1.5, 1–6) for lecturers from the Department, 2.6 (1.5, 1–6) for professors from the Department, 3.0 (1.2, 1–6) for consultant hospital doctors, 3.3 (1.1, 1–6) for non-consultant hospital doctors, 4.5 (1.4, 1–6) for medical students who have recently completed the rotation and 5.0 (1.8, 1–6) for medical students undertaking the rotation.
- The majority responded that *the best time during the rotation to undertake TOSBA* was at regular intervals throughout the rotation (*n* = 77/107, 72 %) rather than in the middle of the rotation only (*n* = 18/107, 17 %), at the end of the rotation only (*n* = 10/107, 9 %), at the start of the rotation only (*n* = 2/107, 2 %) or ‘don’t know’ (*n* = 0/107, 0 %).
- *The use of real patients* rather than simulated patients made the experience ‘much better’ (*n* = 96/107, 90 %) or ‘somewhat better’ (*n* = 10/107, 9 %) as opposed to ‘no difference’ (*n* = 1/107, 1 %), ‘somewhat worse’ (*n* = 0/107, 0 %), ‘much worse’ (*n* = 0/107, 0 %) or ‘don’t know’ (*n* = 0/107, 0 %).
- The majority responded that *the observation of the performance other students and their feedback* made the experience ‘much better’ (*n* = 52/107, 48 %) or ‘somewhat better’ (*n* = 47/107, 44 %) as opposed to ‘no difference’ (*n* = 5/107, 5 %), ‘much worse’ (*n* = 2/107, 2 %), ‘don’t know’ (*n* = 1/107, 1 %) or ‘somewhat worse’ (*n* = 0/107, 0 %).
- The majority responded that feedback should be both verbal and written (n = 85/100, 85 %) rather than verbal at the time of TOSBA only (n = 5/100, 5 %) or ‘don’t know’ (n = 10/100, 10 %). With regard to the feedback of scores, the majority responded that scores should be provided as part of the written feedback shortly after TOSBA is completed (n = 80/107, 74 %) rather as part of the verbal feedback at the time of the TOSBA (n = 18/107, 17 %), after the rotation is completed (n = 8/107, 8 %) or ‘don’t know’ (n = 1/107, 1 %).
- Opinion regarding *the provision of academic credit* was divided with 60 students (*n* = 60/107, 56 %) responding that credit should be given and 46 students (*n* = 46/107, 43 %) responding that academic credit should not be given and 1 student responding ‘don’t know’ (*n* = 1/107, 1 %).

### Student survey – qualitative questions

The majority of students who completed the survey provided free-text comments: 74 students (*n* = 74/107, 69 %) provided free-text comments for ‘what worked well about TOSBA’ and 73 students (*n* = 73/107, 68 %) provided free-text comments about ‘how TOSBA could be improved’. The key themes that emerged from the content analysis (with selected student quotes) are provided in Table 4.

**Table 4:** Themes identified by students regarding TOSBA with selected student quotes

What worked well about TOSBA	What could be improved about TOSBA
• The opportunity to be supervised and receive feedback.	• The provision of more feedback particularly written.
*‘The feedback provided. Allows students to improve in areas before the end of rotation exam.’*	*‘Not enough feedback. I would appreciate some constructive criticism.’*
• It enabled application of theoretical knowledge and skills.	• The identification of topics in advance.
*‘It allowed for critical clinical thinking with background knowledge to be applied in a ‘real life’ setting, which is in essence replicating what will be asked of us once we qualify.’*	*‘Having an overall structure for the 8 weeks would be good i.e. having a set topic list to cover – this way you could better prepare and get more out of the TOSBA.’*
• Its clinical focus and use of real patients.	• More distribution of TOSBA during the rotation.
*‘The roles of differential diagnosis, management, investigations etc. were excellent as they required you to put your theory into practice and apply it to the patient in front of you, instead of learning off reams of information from a book, it made you think like a doctor instead of a student.’*	*‘I had almost all my TOSBAs at the beginning of the rotation with very little knowledge, making it difficult to discuss investigations / management. If they had been a little more evenly spaced we could have time to improve.’*
• It highlighted the standards expected and was a gauge of learning.	
*‘Gave an idea of what was expected of students.’*	
• Its interactive learning approach.	
*‘It is a forum that is discussion based- so for me the learning style was perfect.’*	
• The ability to learn from other students.	
*‘Small group guided learning allowed for exposure to other students’ strengths and made it possible to apply these qualities to your own work.’*	
• Its encouragement of learning throughout the rotation. *‘Continual learning.’*	
• The quality and variety of facilitators.	
*‘The variety of instructors was great, gave you a different perspective on topics.’*	

### Relationship between TOSBA scores and summative examination scores

Of the 157 students who completed TOSBA and the summative examination, the mean number (standard deviation, range) of TOSBA grades awarded per student (out of a possible maximum of 5) were: 1.1 (1.1, 0–5) ‘Good’ grades, 3.0 (1.2, 0–5) ‘Satisfactory’ grades and 0.9 (1.2, 0–4) ‘Poor’ grades. Using these individual grades, students were classified into 1 of 4 TOSBA performance categories as follows: 16 students (*n* = 16/157, 10 %) were classified as ‘Overall Excellent’, 57 students (*n* = 57/157, 36 %) were classified as ‘Overall Good’, 63 students (*n* = 63/157, 40 %) were classified as ‘Overall Satisfactory’ and 21 students (*n* = 21/157, 14 %) were classified as ‘Overall Poor’.

The mean summative examination score (standard deviation, range) was 59.0 % (4.9, 47–71). The mean scores (standard deviation, range) for the individual summative examination components were: OSCE 57.7 % (5.5, 44–70), SBA 58.4 % (7.4, 35–70), SAQ 60.1 % (3.9, 41–81) and LCCE 59.5 % (8.4, 41–81). Of the 157 students who completed the summative examination, 71 students (45 %) passed, 79 students (50 %) passed with a distinction (‘honours’) and 7 students (5 %) failed.

Table 5 shows the summative examination outcomes for students in each TOSBA category. There were 2 main findings. Firstly, TOSBA performance corresponded with summative examination performance in general with a significantly higher number of distinctions in the ‘Overall Excellent’ and ‘Overall Good’ categories compared to ‘Overall Satisfactory’ and ‘Overall Poor’ categories (*p* = 0.003). Secondly, almost all of the students who had been rated as ‘Overall Poor’ on their TOSBA performances (*n* = 20/21, 95 %) subsequently passed the summative examination. Conversely, the majority of students who failed the examination (*n* = 6/7, 86 %) had been rated as ‘Overall Satisfactory’ or ‘Overall Good’.

**Table 5:** Outcome for students by TOSBA categorisation

TOSBA category	Summative examination outcome				*P* value
	Summative score mean standard deviation range	Students who failed	Students who passed	Students who passed with distinction	
All Students *n =* 157 (100 %)	59.0 4.9 47–71	7 (5 %)	71 (45 %)	79 (50 %)	
Poor *n* = 21 (14 %)	55.8 4.6 47–65	1 (5 %)	14 (67 %)	6 (28 %)	0.003
Satisfactory *n* = 63 (40 %)	57.6 4.3 47–66	4 (6 %)	36 (57 %)	23 (37 %)	
Good *n* = 57 (36 %)	60.7 4.5 50–71	2 (4 %)	18 (31 %)	37 (65 %)	
Excellent *n* = 16 (10 %)	62.8 4.2 58–70	0 (0 %)	3 (19 %)	13 (81 %)	

## Discussion

### Principal findings

Students reported that TOSBA was a beneficial learning experience with a positive impact on clinical, communication and reasoning skills. Students rated the quality of feedback provided by TOSBA as high. Students identified the observation of the performance and feedback of other students within their TOSBA team as a key feature. TOSBA performance corresponded with summative examination performance in general. High achieving students performed well in both TOSBA and summative assessments. The majority of students who performed poorly in TOSBA subsequently passed the summative assessments. Conversely, the majority of students who failed the summative assessments had satisfactory scores in TOSBA. There are a number of key questions that arise for medical educators considering the introduction of TOSBA to undergraduate clinical rotations: Does TOSBA improve clinical, communication and/or reasoning skills? Does TOSBA provide quality feedback? Does TOSBA provide a valid and/or reliable assessment? The discussion will consider the current evidence and the findings of this study for each of these questions.

#### Does TOSBA improve clinical, communication and/or reasoning skills?

The positive student responses to questions on their learning from TOSBA and the improved summative examination performance of students who had performed poorly in TOSBA suggest that TOSBA facilitates the development of clinical, communication and reasoning skills among medical students. Medical educators strive to promote the development of clinical, communication and reasoning (critical thinking) skills in their educational programmes [11]. However, the teaching of these skills, particularly higher-order cognitive skills, is challenging [6]. There are many tools described for the direct observation and assessment of clinical skills but there is little data on their use as formative assessment [5]. The successful use of the mini-CEX (mini clinical evaluation exercise) as formative assessment has been described [12]. The enhancement of learning in a 14-week medical clerkship through feedback on student performance at ‘blinded patient encounters’ has been demonstrated [13]. The benefits of a summary assessment tool for formative assessment during clinical rotations has been highlighted [14]. The original TOSBA study performed in medicine and surgery found that students rated TOSBA as a useful learning experience [7, 8]. Similarly, the overwhelming majority of students in this study reported the positive impact of TOSBA on their ability to take and present a history, perform a physical examination, communicate with professionals and, notably, to think critically in relation to patient diagnosis and management. However, students reported that TOSBA had less impact on their ability to communicate with patients or work with other professionals, which may be as a result of the format of TOSBA. Although TOSBA is not intended as a replacement for other teaching methods, it is reassuring that students compared their learning experiences with TOSBA favourably with other teaching methods used during the rotation.

The relationship between TOSBA performance and subsequent summative examination performance provided interesting findings. Students who fail to demonstrate competence in summative examinations are of particular concern to medical educators. They are usually required to undertake a further period of training, often at personal cost to the students themselves and the medical schools [15, 16]. Virtually all students who received poor TOSBA grades subsequently passed the summative examination. It may have been that the feedback from TOSBA encouraged these students to address deficiencies i.e. a ‘wake-up call’. Therefore, TOSBA may have been helpful for this important group of students. Conversely, the majority of students who failed the summative examination performed satisfactorily in TOSBA. In the same way that students rated poorly in TOSBA were given a ‘wake-up call’, some of the students who had performed satisfactorily at TOSBA may have adopted a more complacent approach – an unintended and undesirable consequence. Alternatively, it may be that the facilitators were overly generous in their ratings of these students as it can be challenging for facilitators to provide negative formative assessment for fear it may demotivate their students [17]. However, the fact that the vast majority of students rated as ‘overall poor’ by TOSBA subsequently passed the summative examination (with some achieving a distinction) suggests that falsely reassuring feedback may do a disservice to these students.

#### Does TOSBA provide quality feedback?

The fundamental element of formative assessment is the provision of feedback [4]. The literature has defined the key aspects of effective feedback within medical education [10]. Students rated the quality of their feedback from TOSBA highly, particularly that it used non-judgemental language, was based on directly observed behaviours, was based on clear performance criteria and was clear as to how to apply feedback in practice. There were a number of possible reasons for the high rating of the feedback. Firstly, students undertook a pre-defined clinical task in a structured way rather than the entire patient encounter. This allowed students to prepare for the completion of the pre-defined task (including the identification of the standards expected) and allowed facilitators to provide specific feedback on that task. Secondly, students observed the performance of other students and their feedback. This allowed students to gauge the performance of other students with themselves. This positive effect was highlighted by the survey in which the vast majority of students reported that observing the performance of other students and hearing their feedback made the learning experience better. This echoes the original study and the wider literature where peer learning has been identified as beneficial [7, 11]. Thirdly, the feedback provided by TOSBA was based on interactions with real patients. The literature has emphasised the benefits of experiential learning i.e. the provision of concrete experiences in real clinical settings [18]. Fourthly, quality feedback depends on the experience of the facilitators. In this study the facilitators were experienced clinicians and educators, which likely contributed to the high rating of the feedback by students. This was reinforced by the fact that the students identified the lecturers and professors from the department as the most suitable individuals to facilitate TOSBA. However, students rated other aspects of the feedback lower including the inclusion of their own perceptions and limiting the feedback to 1 or 2 items only. TOSBA tasks are complex and facilitators may have overloaded some students, highlighting the challenge of providing feedback in the clinical environment.

#### Does TOSBA provide a valid and/or reliable assessment?

It is important that robust tools are used for assessment particularly in relation to validity and reliability [19]. A previous study found a moderate positive correlation between TOSBA scores and summative examination scores (*r* = +0.6) [8]. This study did not aim to formally evaluate the validity or reliability of TOSBA. Indeed, an absolute correlation between formative and summative examination results is not expected or even desirable as the purpose of formative assessment is to facilitate learning. However, TOSBA performance corresponded with subsequent summative examination performance in general suggesting that TOSBA was useful at predicting subsequent examination performance.

#### Strengths

The study was undertaken in a well-established medical school with a long tradition of clinical teaching and TOSBA was reported to provide added value to the clinical teaching programme. The study was conducted over a full academic year and all students completed TOSBA, ensuring a diverse mix of student demographics and academic ability. Each student was exposed to a range of staff members, ensuring that the findings were not dependent on a particular facilitator. The study was enhanced by the use of a range of examination methods to evaluate the relationship between TOSBA and subsequent summative examination performance.

#### Limitations

The fact that TOSBA was a new initiative within the O&G programme may have impacted the study: the positive student evaluation may have reflected the novel aspect of the learning method and there may have been a learning curve for the staff members facilitating TOSBA that could have impacted on the quality of feedback provided. The response rate to the student survey was 68 % with a significant difference in some of the demographics between responders and non-responders and therefore the benefit for TOSBA may not have been as marked within some student subgroups. The study focused on student learning experience and academic performance and did not address other important elements: the teaching experience for the staff members facilitating TOSBA and the cost benefit of TOSBA.

#### Implications for academic practice

Formative assessment has been identified as a key focus for reform of higher education institutions in the 21st century [20]. TOSBA provides medical educators with a well-received method of formative assessment in the clinical learning environment. This study also highlights the importance in formative assessment of preventing complacency for students who just meet expectations and the need for accurate feedback (and avoiding false reassurance) to struggling students. In this study TOSBA was used for patients in a ward setting. However, TOSBA could be beneficial within a wide variety of clinical environments such as outpatients or general practice [21]. Future research should address the training of facilitators in providing feedback, the predictive value of TOSBA and the role of early intervention for students identified as struggling by TOSBA.

## Conclusions

TOSBA has a positive impact on the clinical, communication and reasoning skills of medical students through the provision of high-quality feedback. The use of structured pre-defined tasks, the observation of the performance and feedback of other students and the use of real patients are key elements of TOSBA. Avoiding student complacency and providing accurate feedback from TOSBA are on-going challenges.

## Abbreviations

LCCE: Long case clinical examination
Mini-CEX: Mini clinical evaluation exercise
O&G: Obstetrics and Gynaecology
OSCE: Objective Structured Clinical Examination
SAQ: Short answer question
SBA: Single best answer
TCD: Trinity College Dublin
TOSBA: Team Objective Structured Bedside Assessment
